# Transvaginal natural orifice specimen extraction surgery (NOSES) in 3D laparoscopic partial or radical nephrectomy: a preliminary study

**DOI:** 10.1186/s12894-021-00890-9

**Published:** 2021-09-08

**Authors:** Qinxin Zhao, Dongdong Han, Feiya Yang, Sujun Han, Nianzeng Xing

**Affiliations:** 1 Department of Urology, National Cancer Center/National Clinical Research Center for Cancer/Cancer Hospital, Chinese Academy of Medical Sciences and Peking Union Medical College, Beijing, People’s Republic of China; 2 Department of Urology, Beijing Chaoyang Hospital, Capital Medical University, Beijing, People’s Republic of China

**Keywords:** Natural orifice specimen extraction surgery (NOSES), Laparoscopy, Partial nephrectomy, Radical nephrectomy, Renal carcinoma

## Abstract

**Background:**

With the development of minimally invasive technology, more and more people pay attention to aesthetics of the wound after operation. This study is aim to introduce a new surgical technique of transvaginal natural orifice specimen extraction surgery (NOSES) in 3D laparoscopic partial or radical nephrectomy and evaluate the safety, feasibility and clinical effect.

**Methods:**

Eleven patients who underwent 3D laparoscopic partial nephrectomy (n = 7) or radical nephrectomy (n = 4) and NOSES were included in this study. The surgical procedures and techniques, especially the NOSES operation, are reported in detail. In addition, the basic clinical data, perioperative related data, perioperative complications were analyzed.

**Results:**

All 11 patients were performed successfully without conversion to open surgery. The mean total operative time was 133 (84, 150) min. NOSES time was 15 (13, 16) min, and the postoperative hospital stay was 5 (5, 5) d. The mean visual analogue score (VAS) was 3 (2, 4) point and 1 (0, 1) point at 24 h and 48 h after operation, respectively. No patient had recurrence, metastasis and death during the follow-up period of 3 to 17 months. The median Vancouver Scar Scale (VSS) was 1 (1, 1) point. The mean of Female Sexual Function Index (FSFI) was 21.60 (20.20, 21.60), 21.80 (19.80, 21.80) respectively between preoperative and postoperative 3 months, which has no statistical difference (*P* = 0.179). There was no statistical difference in the Pelvic Floor Distress Inventory-short form 20 (PFDI-20) score between preoperative and postoperative 3 months (*P* = 0.142).

**Conclusions:**

Transvaginal NOSES is safe and feasible in 3D laparoscopic partial or radical nephrectomy. Furthermore, it results in low incision-related pain without affecting the pelvic floor and sexual function.

## Background

With the development of laparoscopic technology, more and more patients are pursuing the aesthetic condition of the wound after operation. Laparoscopic radical nephrectomy is still recommended for the operation of renal cancer, and partial nephrectomy or radical nephrectomy is recommended for unilateral renal tumors with T1a–T1b stage or localized tumors [1, 2]. However, the traditional laparoscopic partial nephrectomy or radical nephrectomy are still using the median incision of the lower abdomen to remove specimens, which increase the pain feeling of patients after operation, and affect the wound healing and other related complications. Nowadays natural orifice specimen extraction surgery (NOSES) has attracted wide attention. Compared with the traditional operation, the amount of bleeding, length of hospital stays, and the time of the first postoperative exhaust are significantly better than the traditional laparoscopic specimen extraction [3, 4]. But there are relatively few studies on laparoscopic partial or radical nephrectomy plus transvaginal NOSES. Therefore, since July 2019, our center has performed partial nephrectomy or radical nephrectomy and transvaginal NOSES for 11 female patients and analyzed the relevant data of the patients preoperative and postoperative, and the report is as follows.

## Methods

### Basic clinical data

In this study, 11 patients with renal carcinoma underwent 3D laparoscopic partial nephrectomy or radical nephrectomy and transvaginal NOSES, 7 patients (63.63%) underwent laparoscopic partial nephrectomy and transvaginal NOSES, and 4 patients (36.36%) underwent laparoscopic radical nephrectomy and transvaginal NOSES. All patients were operated on by one experienced urologist. All patients received abdominal magnetic resonance or enhanced computed tomography or abdominal ultrasonography before surgery. Perioperative period, postoperative recovery, complications and oncologic prognosis were recorded. The study was approved by Research Ethics Committee of Cancer Hospital, Chinese Academy of Medical Sciences. All the patients agreed to participate in the study and signed the informed consent. All methods were carried out in accordance with relevant guidelines and regulations.

### Indications and contraindications of NOSES

Indications for surgery: a. Estimate the size of surgical specimens can be taken out from the vagina; b. Married adult female patients; c. Preoperative imaging examination indicates possible renal cancer or renal cancer patients.

Absolute contraindications for surgery: a. Unmarried and infertile women; b. Contractile scar of vagina caused by trauma or surgery; c. Patients with intravaginal infection, cervical neoplasia or carcinogenesis, gynecological pelvic inflammation and other diseases that are not cured before surgery.

Relative contraindications: a. History of pelvic or lower abdominal surgery; b. Obesity.

### Preoperative preparation

Bowel preparation: use glycerol enema machine for enema in the morning before operation; vaginal preparation: use Iodophor for vaginal irrigation 3 days before operation, once a day.

### Surgical methods

#### Preparation steps

**Fig. 1:**
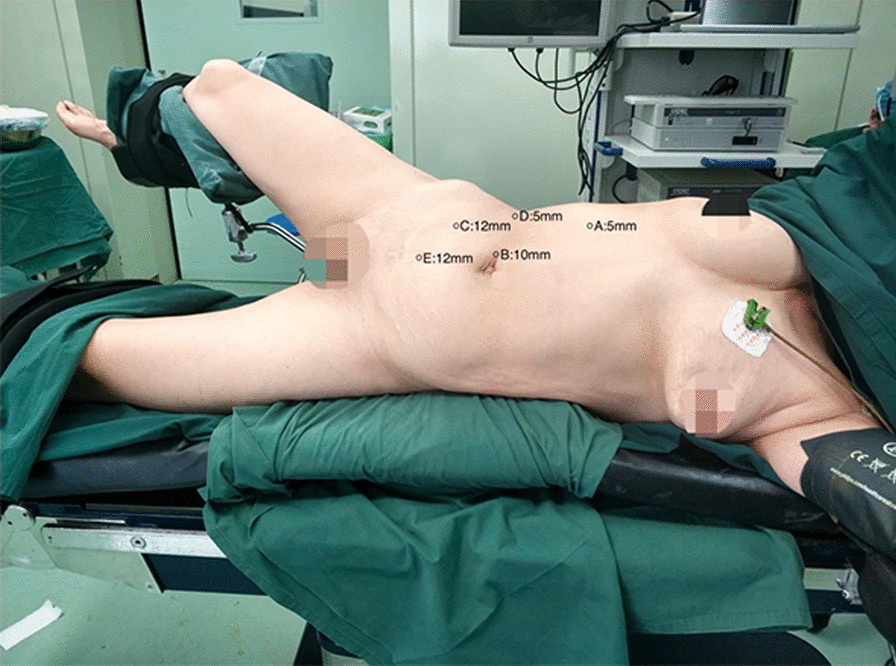
The patient's position and location of trocar. The patient was placed in an oblique position of 70°. The location of trocar: A hole is located under the costal margin of the mid-clavicular line on the affected side, and 5 mm cannula is punctured into the abdominal cavity; B hole is located around the umbilicus and makes a longitudinal incision about 1 cm, and 10 mm Trocar cannula is placed into the laparoscope; C, D and E holes are located at the midpoint of the anterior superior iliac crest and umbilicus line on the affected 12 mm, 12 mm and 5 mm Trocar cannulas were placed at the horizontal intersection of axillary front line and umbilical cord for the placement of operating instruments

Under general anesthesia, the patient was placed in an oblique position of 70°. After disinfection with Iodophor in vagina, a piece of iodophor gauze ball was left to maintain pneumoperitoneal pressure. Figure 1 shows the location of Trocar.

#### Radical nephrectomy

**Fig. 2:**
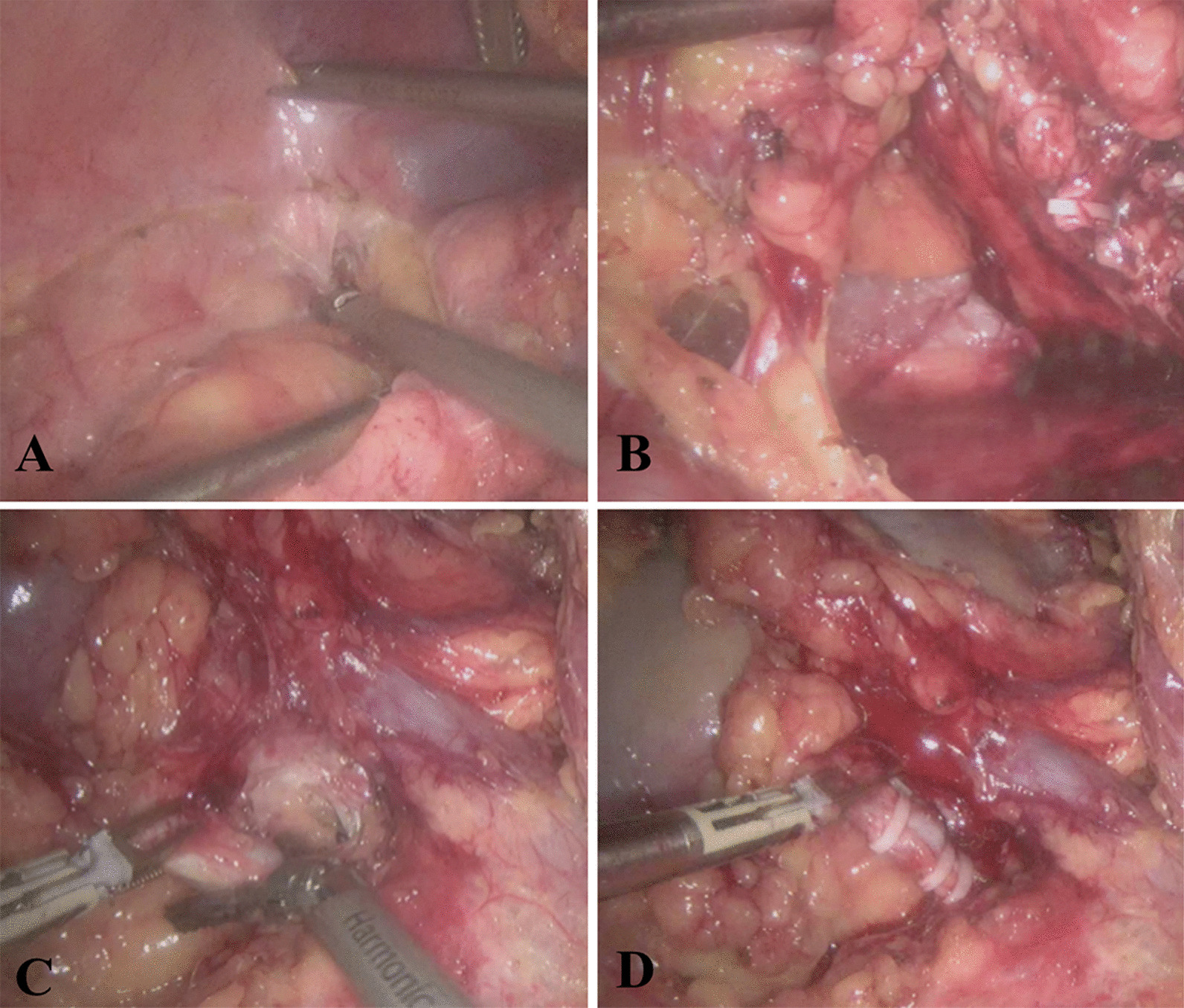
Surgical procedures of laparoscopic radical nephrectomy. **A** Free fascial tissue of colon and abdominal cavity with ultrasound scalpel, and expose retroperitoneal tissue. **B** Peel off the lower part of Gerota fascia with an ultrasonic scalpel, free the ureter, clamp with Hem-o-lock clip and cut off the ureter. **C**, **D** Free along ureter and genital vein toward proximal end, expose renal vein, renal artery behind it, clip renal artery with 3 Hem-o-locks and cut it, renal veins were treated in the same way

a. Exposure of retroperitoneal tissue: Free fascial tissue of colon and abdominal cavity with ultrasound scalpel, and expose retroperitoneal tissue (Fig. 2A). b. Free ureter: Peel off the lower part of Gerota fascia with an ultrasonic scalpel, free the ureter, clamp with Hem-o-lock clip and cut off the ureter (Fig. 2B). c. Free renal artery and vein and ligation, cutting: Free along ureter and genital vein toward proximal end, free and fully expose renal vein, reveal renal artery behind it, clip renal artery with 3 Hem-o-locks and cut it (Fig. 2C), renal veins were treated in the same way (Fig. 2D). d. Preservation of adrenal gland: Peel off the anterior layer of Gerota fascia with an ultrasound scalpel, separate the perirenal fat toward the superior direction, and preserve the adrenal gland. e. Removal of kidney and tumor: Hem-o-lock was used to clamp the small blood vessels and lymphatic vessels around the renal hilum and cut them off. The kidney and tumor were completely free and removed.

#### Partial nephrectomy

**Fig. 3:**
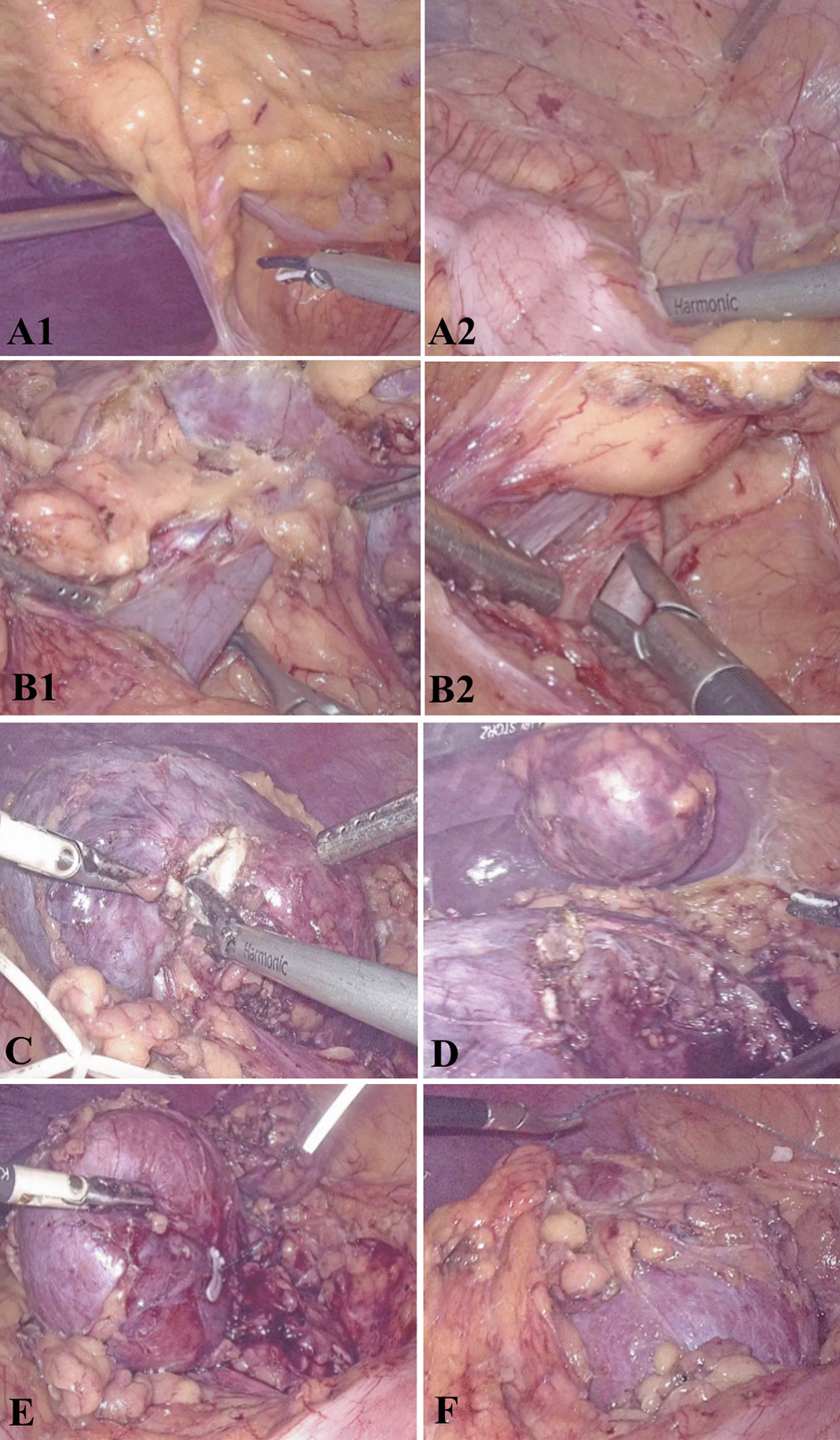
Surgical procedures of laparoscopic partial nephrectomy. a. Exposure of retroperitoneal tissue: Release the fascial tissue of the colon and abdominal cavity with an ultrasound scalpel, turn the hepatic or splenic flexure of the colon to the opposite side, expose the retroperitoneal tissue (**A1-2**). b. Separate the renal artery and vein: Separate the plane of the renal vein along the retroperitoneal and Gerota fascial space, free the renal vein, and find the renal artery behind the renal vein, free the renal artery (**B1-2**). c. Location of the tumor: Cut the perirenal fat capsule in the middle of the kidney to find the location of the tumor, free the adipose tissue around the tumor, expose the tumor at the boundary of normal kidney tissue, preserve the adipose tissue above the tumor (**C**), d. Resect the tumor: Block the main renal artery with vascular clips, and resect the tumor with scissors and aspirator 1 cm outside the edge of the tumor, together with part of normal kidney tissue (**D**). e. Hemostasis and suture: Initial hemostasis using bipolar electrocoagulation on the wound surface. The inner layer was sutured with 3-0 barbed suture, and the renal wound was sutured with 2-0 barbed coil layer, and the suture was fixed intermittently with Hem-o-lock (**E**). f. Restore blood flow and observe blood supply: Loosen the renal artery blocking clamp, observe whether the blood supply, color, elasticity of renal tissue are normal, whether there are obvious bleeding foci, and explore whether the ureter is normal, whether the urine color is normal (**F**). Protein glue can be used to plug the wound to assist hemostasis if necessary, and the perirenal fat capsule can be re-sutured

a. Exposure of retroperitoneal tissue: Release the fascial tissue of the colon and abdominal cavity with an ultrasound scalpel, turn the hepatic or splenic flexure of the colon to the opposite side, expose the retroperitoneal tissue (Fig. 3A1-2). b. Separate the renal artery and vein: Separate the plane of the renal vein along the retroperitoneal and Gerota fascial space, free the renal vein, and find the renal artery behind the renal vein, free the renal artery (Fig. 3B1-2). c. Location of the tumor: Cut the perirenal fat capsule in the middle of the kidney to find the location of the tumor, free the adipose tissue around the tumor, expose the tumor at the boundary of normal kidney tissue, preserve the adipose tissue above the tumor (Fig. 3C), d. Resect the tumor: Block the main renal artery with vascular clips, and resect the tumor with scissors and aspirator 1 cm outside the edge of the tumor, together with part of normal kidney tissue (Fig. 3D). e. Hemostasis and suture: Initial hemostasis using bipolar electrocoagulation on the wound surface. The inner layer was sutured with 3–0 barbed suture, and the renal wound was sutured with 2-0 barbed coil layer, and the suture was fixed intermittently with Hem-o-lock (Fig. 3E). f. Restore blood flow and observe blood supply: Loosen the renal artery blocking clamp, observe whether the blood supply, color, elasticity of renal tissue is normal, whether there are obvious bleeding foci, and explore whether the ureter is normal, whether the urine color is normal (Fig. 3F). Protein glue can be used to plug the wound to assist hemostasis if necessary, and the perirenal fat capsule can be re-sutured.

#### Transvaginal NOSES

**Fig. 4:**
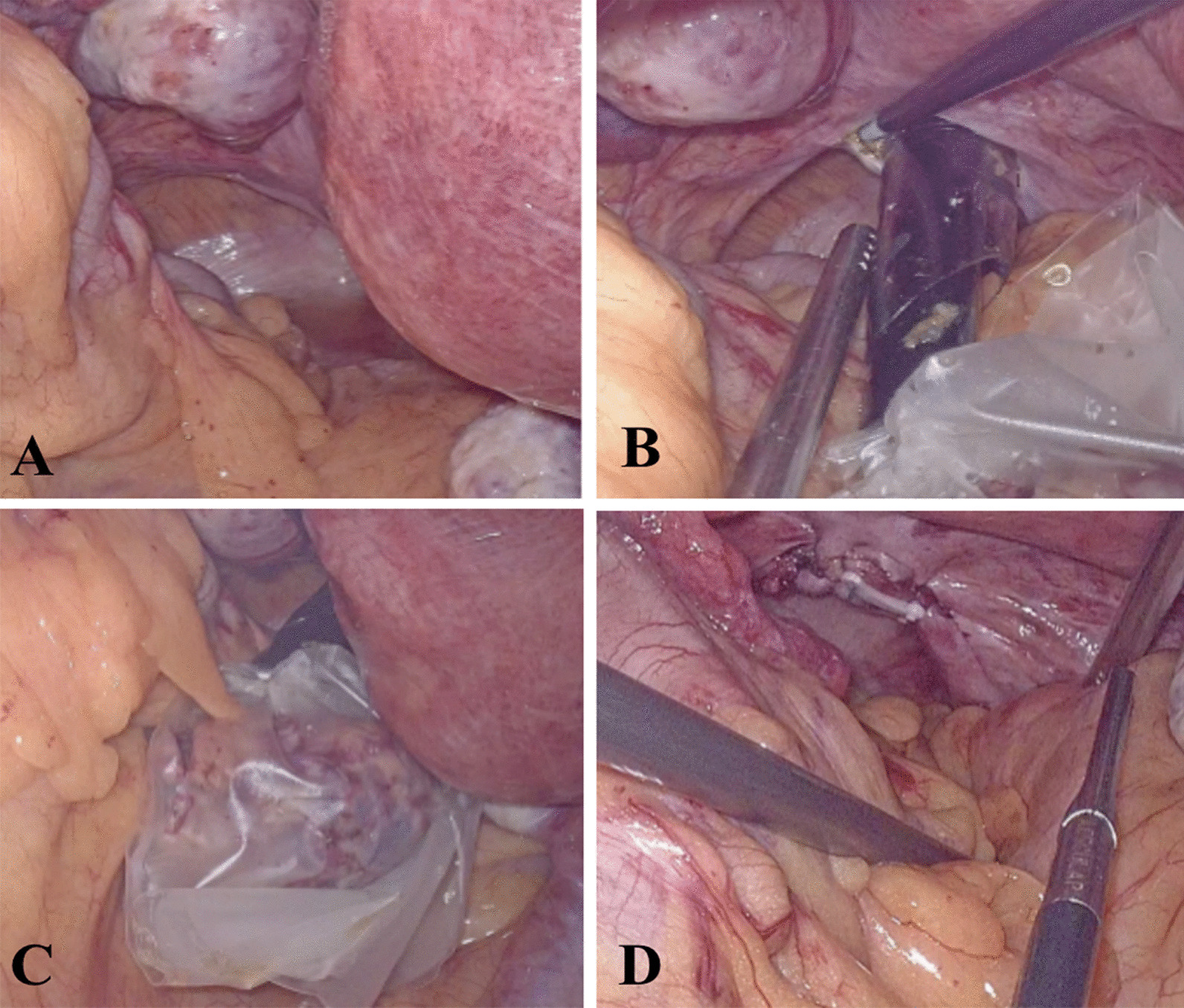
Surgical procedures of laparoscopic transvaginal NOSES. The laparoscopic field of view was transferred to the pelvis, the posterior vaginal fornix was exposed (**A**), 12 mm Trocar was placed in the vagina which had been fully sterilized by gauze strips, and the posterior vaginal fornix was closely attached to the posterior vaginal fornix. The posterior vaginal fornix was transversely cut (**B**) with an electrocoagulation knife to both sides, so that the incision was about 2–3 cm long. The specimen band (**C**) was placed in the transvaginal Trocar, and the excised kidney and part of the ureter were placed into the specimen. Take out the band, hang the vagina with 2-0 barbed thread, and suture the vaginal incision and the posterior fornix exactly (**D**), and suture the lateral peritoneum with 2-0 barbed thread

The laparoscopic field of view was transferred to the pelvis, the posterior vaginal fornix was exposed (Fig. 4A), 12 mm Trocar was placed in the vagina which had been fully sterilized by gauze strips, and the posterior vaginal fornix was closely attached to the posterior vaginal fornix. The posterior vaginal fornix was transversely cut (Fig. 4B) with an electrocoagulation knife to both sides, so that the incision was about 2–3 cm long. The specimen band (Fig. 4C) was placed in the transvaginal Trocar, and the excised kidney and part of the ureter were placed into the specimen. Take out the band, hang the vagina with 2-0 barbed thread, and suture the vaginal incision and the posterior fornix exactly (Fig. 4D), and suture the lateral peritoneum with 2-0 barbed thread.

### Evaluation of surgical effect

Total operation time, estimated intraoperative bleeding volume, intraoperative blood transfusion volume, postoperative creatinine value, postoperative ambulation time, recovery time to resume eating, first time to flatus, postoperative VAS score, postoperative follow-up data (including sexual function, pelvic floor function, vaginal posterior fornix and abdominal incision healing).

### Statistical methods

The data was processed by IBM SPSS statistics v26.0. Continuous data were expressed by means and standard deviation and range. K-S test was used to check whether the preoperative and postoperative data conformed to normal distribution, and t test was used to verify whether the data conformed to normal distribution. Otherwise, rank sum test was used. The test result *P* < 0.05 represented statistical significance.

## Results

All 11 patients were performed successfully without conversion to open surgery. The average of age was 50 (48, 61) years, the average of BMI was 21.3 (23.3, 25.0) kg/m^2^. The Charlson Comorbidity Index (CCI) score was 1 (1, 2) point. The average of American Society of Anesthesiologists (ASA) score was 1 (1, 1) point (Table 1). Three patients (27.27%) had a previous history of abdominal surgery. The mean total operative time was 133 (84, 150). NOSES time was 15 (13, 16) min, and the postoperative hospital stay was 5 (5, 5) d. The time to resume eating was 1 (1, 2) d, and the time to ambulation was 1 (1, 1) d. The time to flatus was 2 (1, 2) d. The mean visual analogue score (VAS) was 3 (2, 4) point and 1 (0, 1) point at 24 h and 48 h after operation, respectively. Postoperative pathological results were clear cell carcinoma in 9 cases (81.82%), chromophobe cell carcinoma in one case (9.09%) and renal angiomyolipoma in one case (9.09%) (Table 2).

**Table 1:** Base information of all 11 patients

Variables	Data
Patient, n	11
Age [median, (IQR)], y	50 (48, 61)
BMI [median, (IQR)], kg/m^2^	21.3 (23.3, 25.0)
CCI [median, (IQR)]	1 (1, 2)
ASA score, n (%)	
1	10 (90.91%)
2	1 (9.09%)
Abdominal surgery history, n (%)	3 (27.27%)
Partial nephrectomy, n (%)	7 (63.63%)
Radical nephrectomy, n (%)	4 (36.36%)

*BMI* body mass index, *CCI* Charlson Comorbidity Index, *ASA* American Society of Anesthesiologists

**Table 2:** Perioperative data of all 11 patients

Variables	Data
Postoperative hospital stays [median, (IQR)], d	5 (5, 5)
Estimated blood lose [median, (IQR)], ml	20 (10, 30)
Transfusion, n (%)	0 (0%)
Total operation time [median, (IQR)], min	133 (84, 150)
NOSES time [median, (IQR)], min	15 (13, 16)
Specimen size [median, (IQR)], cm	6 (4.5, 13)
VAS score [median, (IQR)]	
24 h	3 (2, 4)
48 h	1 (0, 1)
Final pathologic stage	
Clear cell carcinoma, n (%)	9 (81.82%)
Chromophobe cell carcinoma, n (%)	1 (9.09%)
Renal angiomyolipoma, n (%)	1 (9.09%)
T1a	5 (45.45%)
T1b	5 (45.45%)
T2	0 (0%)
T3	1 (9.09%)
T4	0 (0%)
Pathologic stage	
N0	0 (0%)
N1	0 (0%)
N2	0 (0%)
N3	0 (0%)

*NOSES* natural orifice specimen extraction surgery, *VAS* visual analogue scale

The follow-up time was 3–17 months. There were no related complications during the perioperative period. There were no complications such as abdominal distension, constipation, wound pain and so on (Table 3). For the follow-up of pelvic floor function. The mean of preoperative and postoperative 3 months Female Sexual Function Index (FSFI) was 21.60 (20.20, 21.60), 21.80 (19.80, 21.80) respectively, which has no statistical difference (*P* = 0.179). There was no statistical difference in the Pelvic Floor Distress Inventory-short form 20 (PFDI-20) score between preoperative and postoperative 3 months (*P* = 0.142). The median Vancouver Scar Scale (VSS) was 1 (1, 1) point. (Table 4). The trocar wound healed well and was close to the surrounding normal skin tissue, with almost no mark.

**Table 3:** Follow up data of all 11 patients

Variables	Data
Follow-up time [median, (IQR)], m	6 (4, 15)
VSS score [median, (IQR)]	1 (1, 1)
Recurrence, n (%)	0 (0%)
Metastasis, n (%)	0 (0%)
Overall survival, n%	11 (100%)

*VSS* Vancouver Scar Scale

**Table 4:** Preoperative and postoperative sexual function and pelvic floor function contrast of all 11 patients

Variables	Data		
	Preoperative at 3 months [median, (IQR)]	Postoperative at 3 months [median, (IQR)]	*P*
PFDI-20	7 (5, 8)	7 (3, 9)	0.142
FSFI	21.60 (20.20, 21.60)	21.80 (19.80, 21.80)	0.179

*PFDI-20* pelvic floor distress inventory-short form 20, *FSFI* Female Sexual Function Index

## Discussion

With the development of minimally invasive technology, people gradually start to pay more attention to the quality of life and wound aesthetics after surgery. It is well known that laparoscopic partial nephrectomy or radical nephrectomy is currently one of the most effective treatment modalities for renal cancer. But intraoperatively, it is not possible to avoid removing specimens from the abdominal wall incision, consequently increasing postoperative complications such as slower wound healing, increased postoperative pain, and increased infection.

In 1993. Breda et al. [5] reported that the retrieval of specimens from the vaginal natural cavity reduced the disadvantages of extended wound retrieval specimens. However, there is no complete standard and guideline reference for intraperitoneal specimen retrieval in women, so we aimed to do the above studies to explore the feasibility and efficacy of NOSES in 3D laparoscopic partial or radical nephrectomy. Several studies [6, 7] have shown that transvaginal specimen retrieval surgery achieved promising clinical results. In a meta-analysis summarizing 665 patients who underwent surgery via NOSES versus 772 via conventional laparoscopy, patients showed no significant difference in terms of postoperative recurrence rate, postoperative pain, hospital stay, time to first flatus, aesthetic outcome of the wound, and wound infection compared with conventional laparoscopy [8]. But some scholars believe that transvaginal NOSES may cause problems with tumor implantation or wound infection, one meta-analysis concluded that there was no significant difference in oncological outcomes between the NOSES group and the traditional abdominal incision group [9]. Similar results were obtained by Gao et al. [10] who showed no significant difference in 3-year overall survival and disease-free survival between the two groups. In our study, patients did not experience tumor recurrence, implant metastasis within the follow-up date, and also did not experience infection problems in their perioperative wounds, which were more desirable than those of conventional surgery.

Recently, some scholars have used the natural orifice endoscopic surgery (NOTES) in obstetrics and gynecological surgery, urological surgery, and gastrointestinal surgery [11, 12]. However, transvaginal NOTES surgery requires dedicated instruments, and the surgical difficulty is high, with a long learning curve, at present, there are still a small number of developing clinical NOTES at home and abroad, which also makes it difficult to generalize and clinical application [13–15].

The NOSES procedure can be performed without special instruments, and we developed the relevant surgical methods and precautions of transvaginal NOSES in partial nephrectomy or radical nephrectomy based on the above experience. During the operation, we placed a retractable specimen bag, put the specimen into it, and tighten one end of the bag to take the specimen out visually, which can prevent the spread and implantation of tumors. In terms of posterior vaginal vault wound, we use 2-0 barbed suture to avoid fistula and muscle damage in the wound. At the same time, NOSES should also protect the female uterus and adnexa, although the malleability of the female vagina is better, to prevent patients from postoperative pelvic floor dysfunction. We do not recommend NOSES surgery in patients with bulky tumors or already existing metastases. In our study, no patient had vaginal prolapse, labored urination and suffered oncological recurrence. In addition, patients were satisfied with wound healing during the postoperative follow-up time. Most studies [16, 17] have shown that patients do not experience impaired sexual function after transvaginal fornix incision specimen retrieval, and the sexual function of surgical patients, which was consistent with our findings.

## Conclusions

To sum up, transvaginal NOSES is safe and feasible in 3D laparoscopic partial or radical nephrectomy. Furthermore, it results in low incision-related pain without affecting the pelvic floor and sexual function. However, the number of cases is small, and multicenter randomized controlled trials with more cases are needed to further evaluate and verify this conclusion.

## Data Availability

The raw datasets used and/or analyzed during the current study are available from the corresponding author on reasonable request.

## Abbreviations

NOSES: Nature orifice specimen extraction surgery
VAS: Visual analogue score
VSS: Vancouver Scar Scale
FSFI: Female Sexual Function Index
PFDI-20: Pelvic Floor Distress Inventory-short form 20
BMI: Body mass index
CCI: Charlson Comorbidity Index
ASA: American Society of Anesthesiologists
NOTES: Natural orifice endoscopic surgery
